# A model-based hierarchical Bayesian approach to Sholl analysis

**DOI:** 10.1093/bioinformatics/btae156

**Published:** 2024-03-21

**Authors:** Erik VonKaenel, Alexis Feidler, Rebecca Lowery, Katherine Andersh, Tanzy Love, Ania Majewska, Matthew N McCall

**Affiliations:** Department of Biostatistics and Computational Biology, University of Rochester, Rochester, NY 14642, United States; Department of Neuroscience, University of Rochester, Rochester, NY 14642, United States; Department of Neuroscience, University of Rochester, Rochester, NY 14642, United States; Department of Neuroscience, University of Rochester, Rochester, NY 14642, United States; Department of Biostatistics and Computational Biology, University of Rochester, Rochester, NY 14642, United States; Department of Neuroscience, University of Rochester, Rochester, NY 14642, United States; Department of Biostatistics and Computational Biology, University of Rochester, Rochester, NY 14642, United States; Department of Biomedical Genetics, University of Rochester, Rochester, NY 14642, United States

## Abstract

**Motivation:**

Due to the link between microglial morphology and function, morphological changes in microglia are frequently used to identify pathological immune responses in the central nervous system. In the absence of pathology, microglia are responsible for maintaining homeostasis, and their morphology can be indicative of how the healthy brain behaves in the presence of external stimuli and genetic differences. Despite recent interest in high throughput methods for morphological analysis, Sholl analysis is still widely used for quantifying microglia morphology via imaging data. Often, the raw data are naturally hierarchical, minimally including many cells per image and many images per animal. However, existing methods for performing downstream inference on Sholl data rely on truncating this hierarchy so rudimentary statistical testing procedures can be used.

**Results:**

To fill this longstanding gap, we introduce a parametric hierarchical Bayesian model-based approach for analyzing Sholl data, so that inference can be performed without aggressive reduction of otherwise very rich data. We apply our model to real data and perform simulation studies comparing the proposed method with a popular alternative.

**Availability and implementation:**

Software to reproduce the results presented in this article is available at: https://github.com/vonkaenelerik/hierarchical_sholl. An R package implementing the proposed models is available at: https://github.com/vonkaenelerik/ShollBayes.

## 1 Introduction

It has been shown that microglia are key players in countless brain pathologies including neurodegenerative disorders, traumatic brain injury, and psychiatric diseases (Gomez-Nicola and Perry 2015, Prinz *et al.* 2019, Sierra *et al.* 2019). As the main immune cells in the central nervous system, microglia respond to these pathologies in a myriad of ways. Alongside reactive behavior, microglia may also have a direct impact at the onset of several diseases. For example, recent genome-wide association studies showed that genes which are risk factors for Alzheimer’s disease are largely expressed in microglia rather than in other brain cell types (Hemonnot *et al.* 2019). Other specific pathologies which involve the microglia include glioma, strokes, multiple sclerosis, Parkinson’s disease, autism, and schizophrenia (Long-Smith *et al.* 2009, Monji *et al.* 2009, Patel *et al.* 2013, Bogie *et al.* 2014, Takano 2015, Hambardzumyan *et al.* 2016). Further, studies have also linked microglia function to various lifestyle factors such as stress, diet, and alcohol consumption (Tynan *et al.* 2010, Marshall *et al.* 2013, Johnson 2015).

A primary reactive behavior of microglia is to change their morphological phenotype. Homeostatic microglia are ramified cells, characterized by a number of highly branched processes extending from a central soma. In response to the presence of either pathological or physiological stimuli, microglia can reorganize these processes to change their number, shape, and distribution, resulting in a broad spectrum of morphological phenotypes. The morphological landscape of microglia includes a breadth of unique formations, including amoeboid shapes, rod-like shapes, hyper-rammified cells, and bushy (or reactive) cells. Examples of different microglia morphologies can be seen in Figure 1 of Reddaway *et al.* (2023). These morphological changes are a dynamic process which potentially differ depending on the stimulus, environmental context, and the stage of the microglial response (Franco and Fernandez-Suarez 2015, Tang and Le 2016). While it is challenging to make direct inferences about microglial function based purely on morphological changes (Paolicelli *et al.* 2022), morphology remains an important indicator of changes in microglial function in many different physiological and pathological settings.

**Figure 1.**
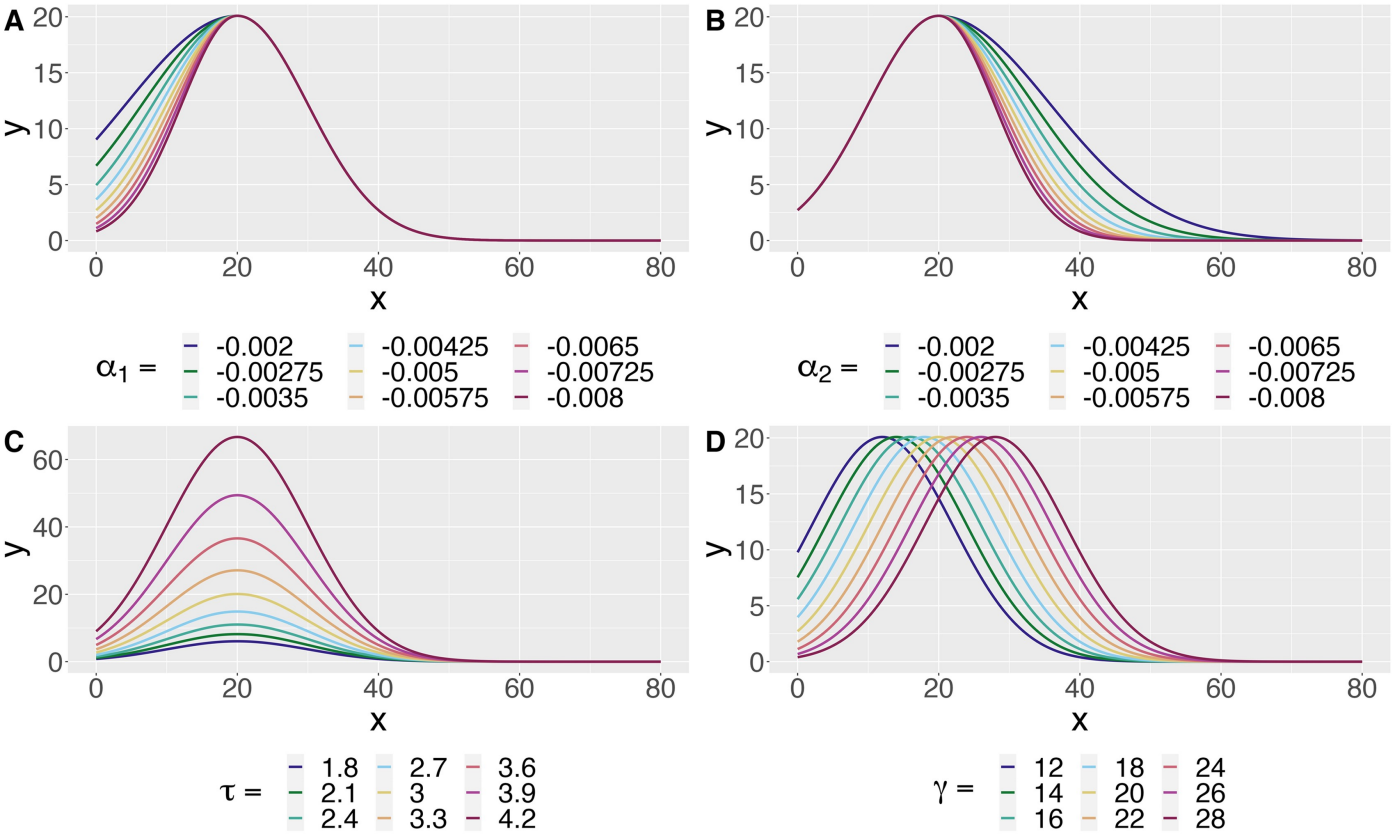
The mean model induced by Equation (1) as each parameter varies. (A) The growth parameter $\alpha_{1}$ controls the behavior of the curve before the change-point. (B) The decay parameter $\alpha_{2}$ controls the behavior of the curve after the change-point. (D) The parameter τ controls the branch maximum of the fitted curve via $e^{\tau }$. (C) The parameter γ controls the critical value, i.e. the change-point, of the fitted curve.

Initially, microglia morphology was analyzed in two dimensions using the concentric shell method (Sholl 1953), now more commonly known as Sholl analysis. The idea of Sholl analysis is to capture cell morphology by estimating the number of branches any given distance away from the soma centeroid. As computational resources have improved, fully 3D methods for capturing and quantifying microglia morphology have been developed which can fully leverage information present in common imaging techniques, such as confocal microscopy. While 3D Sholl analysis is available in commonly used software distributions, these methods often involve estimating a morphological summary metric, such as sphericity, branching numbers, fractal dimension, etc. Each of these metrics attempt to capture a different, biologically relevant aspect of microglia morphology. While these methods are gaining popularity, Sholl analysis remains an important piece of the morphological profile which can be constructed with modern methods.

There has been also been interest in high-throughput methods for analyzing microglial morphology (Heindl *et al.* 2018, Colombo *et al.* 2022). That said, many studies often rely on simple analysis methods implemented in freely available software, such as Sholl analysis, to quantify cell morphology. Despite the popularity of Sholl analysis, methods for performing inference on cell morphology using Sholl data are extremely limited. While Sholl analysis is able to capture a wide range of morphological changes, current methods struggle to take advantage of all available information. We fill this gap by proposing novel methods for performing inference using Sholl data.

## 2 Materials and methods

### 2.1 Existing methods

To perform Sholl analysis, one constructs concentric circles colored (or spheres in three dimensions) around the soma of a cell, the smallest containing the soma, and the largest containing the entire process arbor. Then, the number of times any process crosses each circle is counted. A Sholl curve is constructed by plotting the counts for each circle against the corresponding radii.

As previously mentioned, morphological analysis methods based on Sholl curves are still widely used. Existing methods involve aggressive data reduction or transformation so that basic statistical procedures can be used. Specifically, we typically have access to many cell images in some nested hierarchical structure induced by experimental design, yet the data are often collapsed at the subject level so that each subject only has a single, aggregated Sholl curve. The aggregate curve is usually the point-wise mean of each cell-level curve associated with that subject. Then inference is performed on scalar summaries of the aggregate Sholl curves, for example, the *x* or *y* value of the curve maximum, and ANOVA is used to test for group differences and interaction. For a more detailed discussion of these methods, see the Supplementary Material.

### 2.2 The Sholl curve model

We start by specifying our model for a single Sholl curve. Let $Z_{\geq 0}$ and $R_{\geq 0}$ denote the set of integers and real numbers greater than or equal to 0, respectively. Then a Sholl curve is the pair (*X*, *Y*), where $Y=(y_{1},\ldots ,y_{M})\in Z_{\geq 0}^{M}$ are the process crossings corresponding to the concentric shells of radius $X=(x_{1},\ldots ,x_{M})\in R_{\geq 0}^{M}$, where $x_{1}<\ldots <x_{M}$. Notably, we do not make any requirements that Sholl analysis be performed in two or three dimensions. The model is then given by:

(1) $$\begin{array}{c} \begin{array}{c} y_{i}|x_{i}∼\text{Poisson}(\mu_{i}) \end{array}\\ \begin{array}{c} \;\text{log}(\mu_{i})=\{\begin{array}{c} \alpha_{1}{\left(\gamma \;-\;x_{i}\right)}^{2}\;+\;\tau ,\text{for}\;x_{i}<\gamma \\ \alpha_{2}{\left(x_{i}\;-\;\gamma \right)}^{2}\;+\;\tau ,\;\text{else} \end{array} \end{array} \end{array}$$

where $\alpha_{1},\alpha_{2}<0,\;0<\gamma <x_{M}$, and τ > 0. This is essentially a generalized nonlinear change-point model assuming a Poissonian random component with canonical link function. Intuitively, the log transform of Sholl curves are approximately asymmetric quadratics (Supplementary Fig. S2), which we directly model in the log-mean function. Since Sholl curves are count data, a Poissonian random component with canonical log link is a natural approach.

We think of this model as a combination of a “growth-curve” and a “decay-curve,” which are separated by the change-point γ. The parameters $\alpha_{1}$ and $\alpha_{2}$ control the growth and decay curves, respectively. The maximum of the fitted curve is given by $\left(\gamma ,e^{\tau }\right)$, allowing us to directly estimate the critical value and branch maximum, which are two of the most common Sholl curve summaries. We can also retrieve the *y*-intercept of the estimated curve as $\text{exp}(\alpha_{1}\gamma^{2}+\tau )$, which is interpreted as the expected number of processes originating from the soma. Changes in the mean model as each parameter varies can be seen in Fig. 1.

### 2.3 Hierarchical Sholl curve modeling

Often, we are interested in morphological changes across treatments, conditions, or genotypes, which occur at the highest level of an experimental hierarchy. As previously discussed, current methods aggregate lower-level data (typically via averaging) to enable classic analysis such as ANOVA. The mean model for a single Sholl curve specified in Equation (1) can be embedded in a hierarchical model. Here, we demonstrate this for one of the most common microglial experimental designs: a two-factor model with a potential interaction at the highest level along with lower-level subject data.

To fix ideas, we focus on a specific motivating example dataset, which is a subset of the data from a study investigating the role of microglia in experience-dependent synaptic plasticity (Sipe *et al.* 2016). These data were used to demonstrate that ocular dominance plasticity induces hyper-ramification of microglia and that this effect is limited to the cortical area undergoing plasticity, which is the contralateral binocular visual cortex. Although these data originally contained multiple images and cells per animal, we reanalyze the truncated data using the proposed method to facilitate more direct comparison with the original analysis.

**Figure 2.**
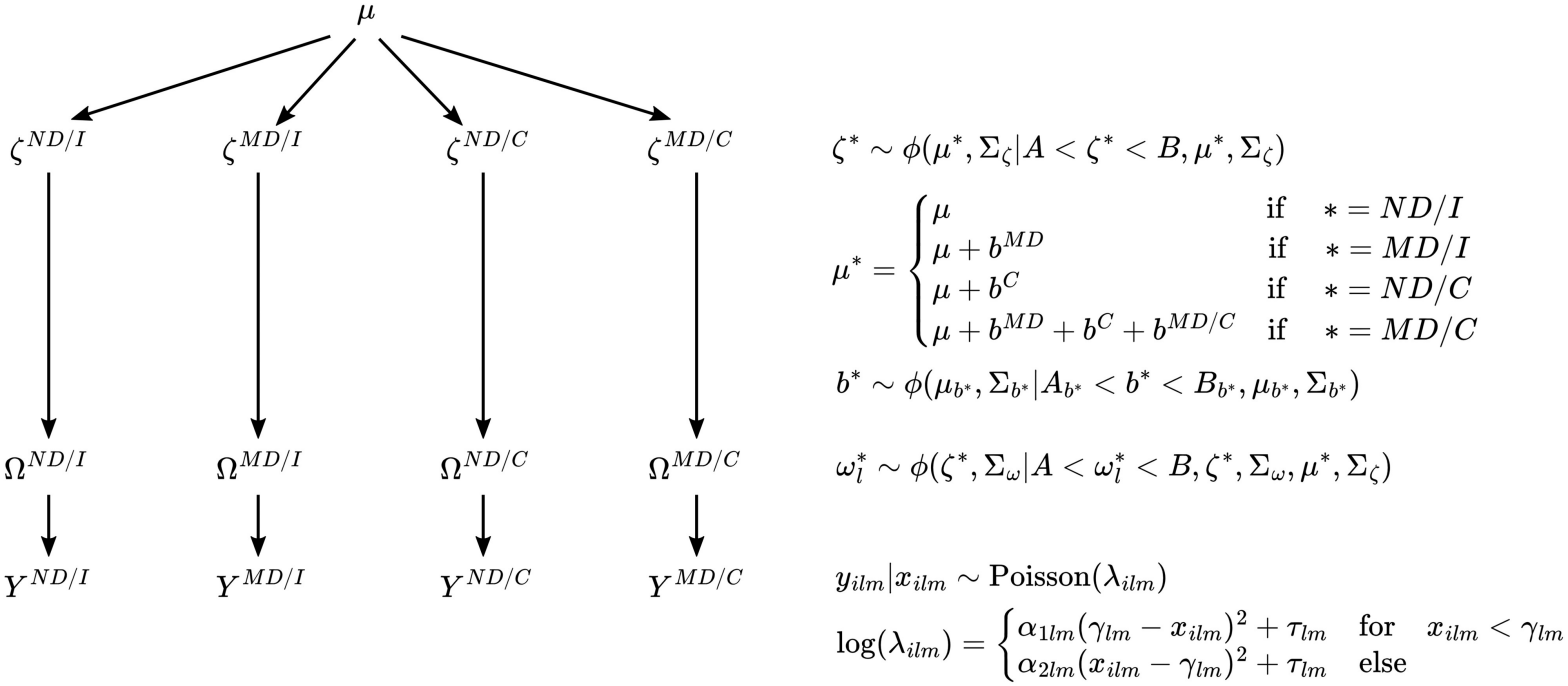
Hierarchical structure for a common experimental design with two binary factors (ND/MD and I/C) and an interaction. We assume parameters at any level are randomly sampled from the corresponding distribution in the next highest level. Here, for some combination of groups ∗, ϕ(∗) denotes the Gaussian distribution, $\zeta^{*}$ denotes group-level parameters, $\Omega^{*}=(\omega_{1}^{*},\ldots ,\omega_{L}^{*})$ denotes animal-level parameters, and $Y^{*}=(y_{1lm},\ldots ,y_{\mathrm{Nlm}})$ denotes Sholl curve process crossings. Additionally, group combination is indexed by *m* and we model group level effects as additive terms $b^{*}$ on the mean parameter for group level distributions. For a given parameter subscript, Σ denotes the corresponding variance parameters for the Gaussian distribution for that parameter. Gaussian priors are truncated via *A* and *B* to enforce the parameter constraints of Equation (1).

These data contain two grouping variables: condition and side. Condition is either monocular deprivation (MD) or no deprivation (ND), and side is either ipsilateral (I) or contralateral (C). We model group and interaction effects for each parameter in Equation (1) so that group-level inference can be performed. We can see the hierarchical structure of the model in Fig. 2. The population-level parameters seen in Fig. 2 are defined as $\mu =(\mu_{\alpha_{1}},\mu_{\alpha_{2}},\mu_{\gamma },\mu_{\tau })$. Denoting groups for the first categorical variable as either ND or MD and the second as either I or C, the overall group level parameter vector is defined as $\zeta^{*}$, where ∗ is replaced with group identifiers. We enable inference at the group level by defining $\zeta^{*}$ as a sum of population level parameters and group effects. Group effects are defined as:

$$\begin{array}{cc} b^{\mathrm{MD}}∼\phi (\mu_{b^{\mathrm{MD}}},\Sigma_{b^{\mathrm{MD}}}|(-\infty ,-\infty ,-\mu_{\gamma },-\mu_{\tau })<b^{\mathrm{MD}}< & \\ \left(-\mu_{\alpha_{1}},-\mu_{\alpha_{2}},\infty ,\infty ),\mu_{b^{\mathrm{MD}}},\Sigma_{b^{\mathrm{MD}}}\right) & \end{array}$$

and

$$\begin{array}{cc} b^{C}∼\phi (\mu_{b^{C}},\Sigma_{b^{C}}\;|(-\infty ,-\infty ,-\mu_{\gamma },-\mu_{\tau })<b^{C}< & \\ \left(-\mu_{\alpha_{1}},-\mu_{\alpha_{2}},\infty ,\infty ),\mu_{b^{C}},\Sigma_{b^{C}}\right) & \end{array}$$

respectively, which are interpreted as a shift in the group mean for the corresponding group. Similarly, the interaction effect is given by:

$$\begin{array}{cc} b^{MD/C}∼\phi (\mu_{b^{MD/C}},\Sigma_{b^{MD/C}} & |A_{b^{MD/C}}<b^{MD/C}<\\ B_{b^{MD/C}},\mu_{b^{MD/C}},\Sigma_{b^{MD/C}}) & \end{array}$$

where

$$\begin{array}{cc} A_{b^{\mathrm{MD}/C}}=(-\infty ,-\infty ,-(\mu_{\gamma }\;+\;b_{\gamma }^{\mathrm{MD}}\;+\;b_{\gamma }^{C}),-(\mu_{\tau }\;+\;b_{\tau }^{\mathrm{MD}}\;+\;b_{\tau }^{C})) & \\ B_{b^{\mathrm{MD}/C}}=(-(\mu_{\alpha_{1}}\;+\;b_{\alpha_{1}}^{\mathrm{MD}}\;+\;b_{\alpha_{1}}^{C}),-(\mu_{\alpha_{2}}\;+\;b_{\alpha_{2}}^{\mathrm{MD}}\;+\;b_{\alpha_{2}}^{C}),\infty ,\infty ), & \end{array}$$

which is interpreted as a shift in the group mean for group MD/C. The bounds on these effects are set to constrain the group level means within (*A*, *B*), i.e. the support of the truncated normal distributions. The parameter vector for animal *l* in group ∗ are denoted by $\omega_{l}^{*}$, with $\Omega^{*}=\{\omega_{l}^{*}:l\in (1,\ldots ,L)\}$.

Truncated normal priors are assumed for population and animal level parameters. We truncate normal distributions ϕ at each level of the hierarchy to enforce the parameter space constraints of Equation (1). The lower bound of the parameter space is A=(−∞,−∞,0,0) and the upper bound is $B=(0,0,\tilde{x},\infty )$, where $\tilde{x}$ is the least upper bound on the support of the Sholl curves. Similarly, we assume truncated normal hyper-priors on mean parameters for each effect, where the truncation is identical to the corresponding effect bounds. As before, we assume half-t priors on all standard deviation parameters in the model, including half-t hyper-priors for the effect standard deviations.

The model is fit using MCMC via rjags. Details regarding the sampling procedure along with model diagnostics are found in the Supplementary Material.

### 2.4 Simulation study

We limit simulation to the model discussed in Section 2.3, except we incorporate an additional level in the hierarchy after animal corresponding to cell-level data. We simulate data under six scenarios, primarily considering changes for effects on τ at the group level since it corresponds to the most common Sholl curve summary, i.e. the branch maximum. For details regarding simulation scenarios, see the Supplementary Material.

## 3 Results/application

### 3.1 MD/ND dataset

To examine whether microglia play a role in the process of experience-dependent synaptic plasticity, investigators previously assessed microglial response by assaying changes in microglial morphology after inducing ocular dominance plasticity (Sipe *et al.* 2016). Tissue sections were generated from wildtype mice that had been monocularly deprived via eyelid suturing for 12 h. Sections underwent histology for a microglia-specific marker (Iba-1) and images of the binocular primary visual cortex were generated using confocal microscopy at 40× magnification in both brain hemispheres to include visual areas both contralateral and ipsilateral to the deprived eye. Z-stacks taken at 1 micron z-step were Gaussian filtered and compressed into a single z-projection. Microglia whose entire process arbor was contained within the image were individually selected and cropped into a new image. Each image was thresholded to generate a binarized outline of the process arbor and filtered to remove artefacts before performing Sholl analysis. Analysis of these data was performed by constructing animal level aggregate Sholl curves and fitting an ANOVA at each radius of the aggregate curves, which were used to test differences in process crossings between experimental conditions in both cortical hemispheres. We applied our hierarchical modeling approach to these data as described in the Methods.

**Figure 3.**
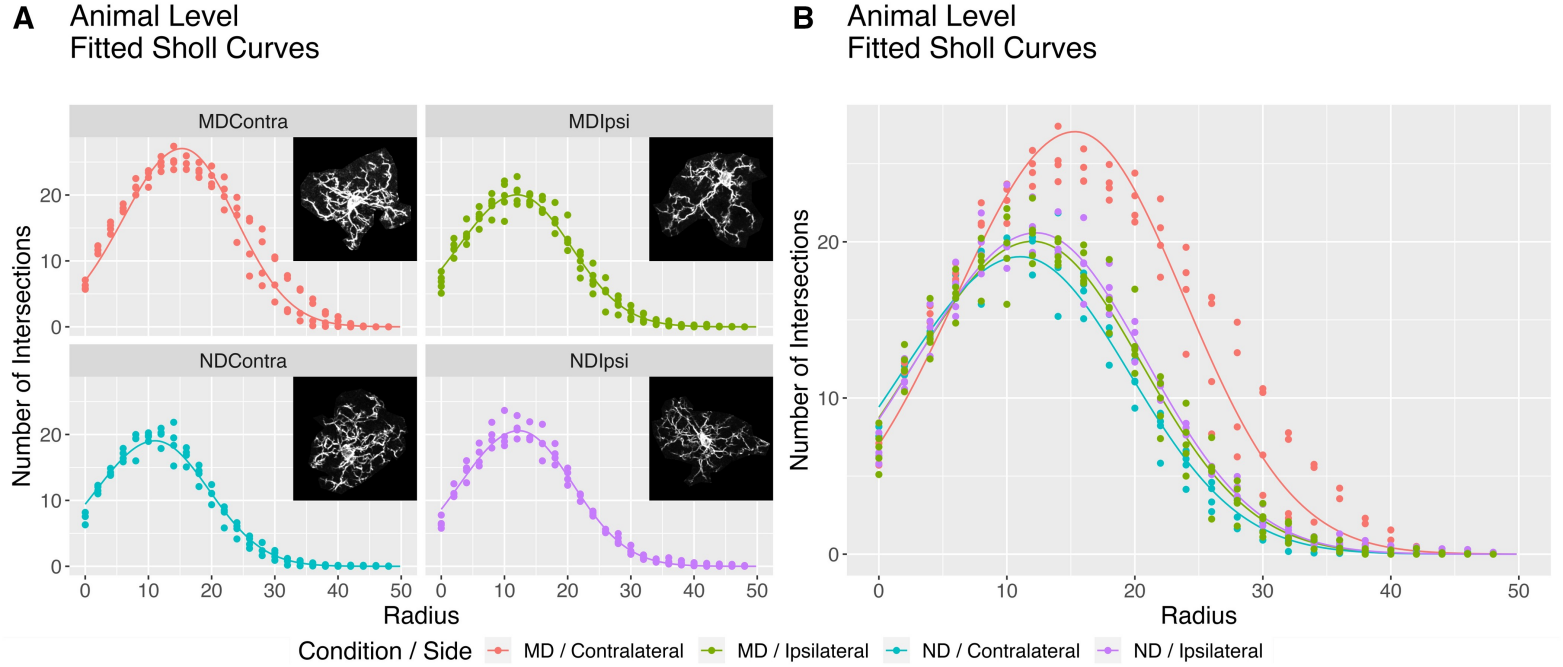
Group-level fitted curves obtained by fitting model 2 to the MD/ND dataset. (A) Fitted curves faceted by group, superimposed over animal level Sholl curves. One microglia from each group is superimposed over the corresponding panel. (B) All four facets from panel (A), superimposed to better show hyper-ramification of the MD/Contra group.

**Figure 4.**
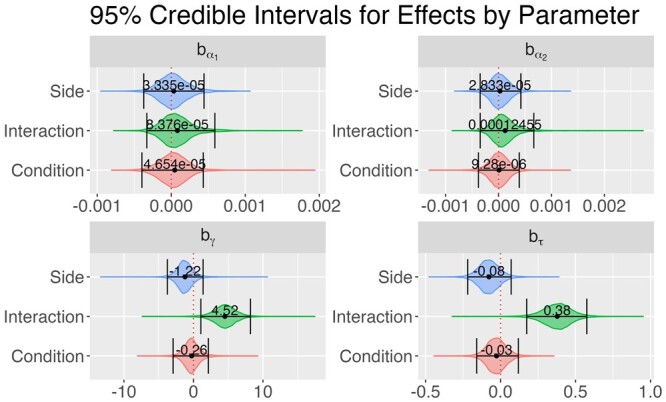
95% credible intervals for each effect in the MD/ND dataset, computed as the highest density posterior interval. Credible intervals are superimposed over the approximate posterior distributions obtained via MCMC. Estimated posterior means are represented by black dots with point estimates displayed above. The dotted vertical line is fixed at zero.

The fitted curves from our model capture the Sholl curve structure well (Fig. 3A). The curve for group MD/C is quite large relative to the other groups (Fig. 3B), indicating potential hyper-ramification of cells in this group. We show 95% credible intervals for group and interaction effects in Fig. 4. These are superimposed over the approximate posterior density obtained via MCMC. Most parameters have posterior mass roughly centered at zero, with the exception of interaction effects on τ and γ. Clearly most of the posterior mass for both these effects falls above zero, which indicates a positive interaction effect associated with these parameters. To perform formal inference, we define a cutoff of 0.95, and check if an effect has at least 95% of its posterior mass either above or below zero. Table 1 shows the estimated posterior probability each effect is less than zero. We say an effect exists in the positive direction for a parameter if this estimate is less than 0.05. Conversely, an effect exists in the negative direction if this estimate is more than 0.95. With respect to this cutoff, we can say a positive interaction effect exists for both τ and γ, while no other effects meet this criterion. Recall that the branch maximum and critical value are given by $e^{\tau }$ and γ in our model, respectively. This suggests microglia hyper-ramification is indeed limited to the contralateral binocular visual cortex, which agrees with the original analysis of these data.

**Table 1.** Estimated posterior probability of a negative effect for each parameter in the MD/ND model.^a^

	$\hat{P}(\text{Effect}<0)$		
Parameter	Condition	Side	Interaction
$\alpha_{1}$	0.413	0.392	0.333
$\alpha_{2}$	0.487	0.456	0.321
γ	0.604	0.853	0.020
τ	0.682	0.873	0.002

a Quantities are estimated as the proportion of MCMC samples that fall below 0.

### 3.2 Application to additional datasets

We applied our method to two additional datasets with different experimental designs. These applications are described in detail in the Supplementary Material. The first example, which we refer to as the Ungrouped Mouse Dataset (Supplementary Material S3.1), demonstrates how sources of uncertainty in a standard experimental setting are propagated through the model in a collection of replicate animals. The second example, which we refer to as the GPNMD Knockout Dataset (Supplementary Material S3.2), demonstrates how we can incorporate effects at various levels of the experimental hierarchy, while also testing an interaction and directly modeling cell level data. These datasets were chosen to represent to additional common experimental designs and can serve as a blueprint for applying our method to other data from the same or a similar design.

### 3.3 Simulation study

To simplify notation, we adopt the language of the applied example (see Section 3) for grouping variables and corresponding effects. In Table 2, we report the frequentist power and FPR estimates for the ANOVA at the 0.05 level. Also reported in Table 2 are ${\hat{p}}_{<0}$ and ${\hat{p}}_{>0}$ for the proposed method, which can be thought of as a Bayesian equivalent (see Supplementary Material for details). In scenario 1, when there is no true effect, both methods perform comparably. In scenario 2, when an effect of +0.5 is introduced to the “condition” group, ${\hat{p}}_{>0}$ is almost always 1. Additionally, we also observe ${\hat{p}}_{>0}$ almost always equal to 1 when we introduce an interaction effect of +0.5. When an effect of −0.25 is introduced to the “side” group, we see slightly smaller values for ${\hat{p}}_{<0}$, which are ∼0.92 and 0.80 in scenarios 3 and 4, respectively. In scenario 5, when cells per animal is increased, we see ${\hat{p}}_{<0}$ for side increase to 0.94. Our method seems to struggle when variance terms are large relative to the effect size. Specifically, in scenario 6 when the side effect is −0.25 and the animal-level variance is increased from $0.1^{2}$ to $0.25^{2}$, we see ${\hat{p}}_{<0}$ for side and ${\hat{p}}_{>0}$ for interaction dip to 0.38 and 0.72, respectively. In contrast, the ANOVA-based method, while fully powered in some scenarios, struggles when more effects and data are introduced. This is apparent in scenario 4, where ANOVA struggles to detect the side effect in the presence of both condition and interaction effects. Additionally, when the cells per animal is increased in scenario 5, we actually see a decrease in power to detect the side effect relative to scenario 4. In scenario 6, ANOVA reports a power of 0.14 for detecting the side effect, which is not much larger than the FPR of 0.12 when no effect is present. Overall, the proposed method performs favorably across scenarios when true effects are present, while, at worse, performing comparably to ANOVA at controlling the false discovery rate.

**Table 2.** Estimated type-I error rate (FPR) and power for ANOVA, compared with ${\hat{p}}_{<0}$ and ${\hat{p}}_{>0}$ for the proposed model under each simulation scenario.^a^

		Method											
		Proposed						ANOVA					
		Condition		Side		Interaction		Condition		Side		Interaction	
Scenario	Parameter	${\hat{p}}_{<0}$	${\hat{p}}_{>0}$	${\hat{p}}_{<0}$	${\hat{p}}_{>0}$	${\hat{p}}_{<0}$	${\hat{p}}_{>0}$	Power	FPR	Power	FPR	Power	FPR
1	$\alpha_{1}$	0.04	0.00	0.00	0.02	0.00	0.00	—	—	—	—	—	—
	$\alpha_{2}$	0.02	0.02	0.06	0.02	0.00	0.00	—	—	—	—	—	—
	γ	0.04	0.08	0.02	0.02	0.04	0.00	—	0.06	—	0.06	—	0.06
	τ	0.10	0.02	0.08	0.06	0.04	0.14	—	0.12	—	0.12	—	0.16
2	$\alpha_{1}$	0.00	0.02	0.00	0.00	0.00	0.00	—	—	—	—	—	—
	$\alpha_{2}$	0.02	0.02	0.02	0.00	0.00	0.00	—	—	—	—	—	—
	γ	0.04	0.02	0.02	0.00	0.00	0.00	—	0.06	—	0.08	—	0.06
	τ	0.00	1.00	0.04	0.08	0.08	0.06	1.00	—	—	0.18	—	0.14
3	$\alpha_{1}$	0.02	0.00	0.00	0.02	0.00	0.00	—	—	—	—	—	—
	$\alpha_{2}$	0.02	0.02	0.00	0.00	0.00	0.00	—	—	—	—	—	—
	γ	0.02	0.08	0.00	0.04	0.00	0.02	—	0.04	—	0.10	—	0.04
	τ	0.04	0.18	0.92	0.00	0.10	0.06	—	0.22	0.94	—	—	0.18
4	$\alpha_{1}$	0.00	0.02	0.00	0.00	0.00	0.02	—	—	—	—	—	—
	$\alpha_{2}$	0.02	0.00	0.00	0.00	0.00	0.00	—	—	—	—	—	—
	γ	0.00	0.02	0.00	0.02	0.02	0.02	—	0.02	—	0.08	—	0.00
	τ	0.00	1.00	0.80	0.00	0.00	1.00	1.00	—	0.56	—	0.98	—
5	$\alpha_{1}$	0.02	0.00	0.00	0.00	0.00	0.00	—	—	—	—	—	—
	$\alpha_{2}$	0.02	0.00	0.00	0.02	0.00	0.00	—	—	—	—	—	—
	γ	0.02	0.02	0.00	0.08	0.00	0.02	—	0.06	—	0.02	—	0.12
	τ	0.00	1.00	0.94	0.00	0.00	1.00	1.00	—	0.34	—	0.94	—
6	$\alpha_{1}$	0.00	0.00	0.00	0.00	0.00	0.00	—	—	—	—	—	—
	$\alpha_{2}$	0.00	0.04	0.02	0.02	0.00	0.00	—	—	—	—	—	—
	γ	0.02	0.08	0.00	0.08	0.04	0.02	—	0.06	—	0.02	—	0.12
	τ	0.00	0.96	0.38	0.00	0.00	0.72	1.00	—	0.14	—	0.44	—

a Power and FPR for the ANOVA model are estimated at the 0.05 level of significance. Equations for computation of ${\hat{p}}_{<0}$ and ${\hat{p}}_{>0}$ for the proposed model are displayed in the Supplementary Material. Dashes fill cells where there is no relevant quantity.

## 4 Discussion

Sholl analysis is still one of the primary tools used in the morphometric analysis of microglia. We proposed a model for directly modeling Sholl curves, filling a long existing gap in the morphological inference pipeline. We generalized this model to a hierarchical Bayesian framework which naturally captures the nested structure of microglia imaging datasets. We applied our model to real data and compared the proposed model to the analysis method previously applied to these data via simulation study.

Our applied examples showcase the flexibility of our method in capturing the myriad of shapes Sholl curves can take. We also demonstrated our model’s ability to capture relevant effects, potentially existing at multiple levels of the hierarchy. In our simulation study, we showed the proposed method performs well when true effects are present, while being comparable to the competing method at controlling false discovery. In comparison, the ANOVA-based method can be fully powered when a large enough effect exists but can become problematic as more data below the level of truncation is made available, in the presence of many true effects, or when the effect size is too small.

Our method can have some trouble sampling $\alpha_{1}$ and associate effects. Often, we see low effective sample size relative to other parameters, along with difficulty getting chains to converge. The latter is reflected in the approximate posterior for $\alpha_{1}$ effects in Supplementary Fig. S7. This could be alleviated by reparameterizing the model. Instead of modeling the growth curve with $\alpha_{1}$, we can instead model the y-intercept directly. This may even be the preferred parameterization if estimating the number of processes originating from the soma is of interest. Our simulation study was also limited to effects on τ, leaving the door open for more rigorous study of other model parameters.

There are several ways this model can be further generalized. The hierarchical framework can be relaxed to allow separate variance parameters within each level. Additionally, Equation (1) can be generalized by including nonlinear parameters $\kappa_{1}$ and $\kappa_{2}$ via

$$\begin{array}{c} \;\text{log}\;\mu_{i}=\{\begin{array}{c} \alpha_{1}{\left(\gamma -x_{i}\right)}^{\kappa_{1}}+\;\tau ,\text{for}\;x_{i}<\gamma \\ \alpha_{2}{\left(x_{i}-\gamma \right)}^{\kappa_{2}}\;+\;\tau ,\text{else} \end{array} \end{array}$$

for $\kappa_{1},\kappa_{2}>1$. This mean model allows more flexible characterization of the growth and decay states in Sholl curves.

In summary, we believe Sholl-based morphological analyses can greatly benefit from model-based methods which utilize all available data. Though the applied examples in this article are limited to microglia, Sholl analysis is also a common method for quantifying the morphology of other cells, particularly neurons. We predict that our method is flexible enough to adequately capture the Sholl curve of other cell types, though modifications should be made to the specific model hierarchy to match the experimental design. While no single morphological summary can capture the breadth of possible cell morphologies, Sholl analysis remains a useful method for discovering morphological differences in experimental settings. We developed this method as a step toward more rigorous morphological analysis when Sholl analysis is an applicable method to quantify cell morphology. We anticipate that the proposed methodology will lead to improved analysis of microglial images by uncovering the changes in morphology that are most predictive of alterations in microglial function.

## Supplementary Material

btae156_Supplementary_Data

## Data Availability

The data underlying this article, along with code to reproduce the results presented in this article, are available on GitHub at: https://github.com/vonkaenelerik/hierarchical_sholl. An R package implementing the proposed models is available at: https://github.com/vonkaenelerik/ShollBayes.

## References

[btae156-B1] Bogie JFJ , Stinissen P, Hendriks JJA. Macrophage subsets and microglia in multiple sclerosis. Acta Neuropathol 2014;128:191–213. 24952885 10.1007/s00401-014-1310-2

[btae156-B2] Colombo G , Cubero RJA, Kanari L et al A tool for mapping microglial morphology, morphomics, reveals brain-region and sex-dependent phenotypes. Nat Neurosci 2022;25:1379–93. 36180790 10.1038/s41593-022-01167-6 PMC9534764

[btae156-B3] Franco R , Fernandez-Suarez D. Alternatively activated microglia and macrophages in the central nervous system. Prog Neurobiol 2015;131:65–86. 26067058 10.1016/j.pneurobio.2015.05.003

[btae156-B4] Gomez-Nicola D , Perry VH. Microglial dynamics and role in the healthy and diseased brain: a paradigm of functional plasticity. Neuroscientist 2015;21:169–84. 24722525 10.1177/1073858414530512 PMC4412879

[btae156-B5] Hambardzumyan D , Gutmann DH, Kettenmann H. The role of microglia and macrophages in glioma maintenance and progression. Nat Neurosci 2016;19:20–7. 26713745 10.1038/nn.4185 PMC4876023

[btae156-B6] Heindl S , Gesierich B, Benakis C et al Automated morphological analysis of microglia after stroke. Front Cell Neurosci 2018;12:106. 29725290 10.3389/fncel.2018.00106 PMC5917008

[btae156-B7] Hemonnot A-L , Hua J, Ulmann L et al Microglia in Alzheimer disease: well-known targets and new opportunities. Front Aging Neurosci 2019;11:233. 31543810 10.3389/fnagi.2019.00233 PMC6730262

[btae156-B8] Johnson RW. Feeding the beast: can microglia in the senescent brain be regulated by diet? Brain Behav Immun 2015;43:1–8. 25451610 10.1016/j.bbi.2014.09.022 PMC4258457

[btae156-B9] Long-Smith CM , Sullivan AM, Nolan YM. The influence of microglia on the pathogenesis of Parkinson’s disease. Prog Neurobiol 2009;89:277–87. 19686799 10.1016/j.pneurobio.2009.08.001

[btae156-B10] Marshall SA , McClain JA, Kelso ML et al Microglial activation is not equivalent to neuroinflammation in alcohol-induced neurodegeneration: the importance of microglia phenotype. Neurobiol Dis 2013;54:239–51. 23313316 10.1016/j.nbd.2012.12.016 PMC3629000

[btae156-B11] Monji A , Kato T, Kanba S. Cytokines and schizophrenia: microglia hypothesis of schizophrenia. Psychiatry Clin Neurosci 2009;63:257–65. 19579286 10.1111/j.1440-1819.2009.01945.x

[btae156-B12] Paolicelli RC , Sierra A, Stevens B et al Microglia states and nomenclature: a field at its crossroads. Neuron 2022;110:3458–83. 36327895 10.1016/j.neuron.2022.10.020 PMC9999291

[btae156-B13] Patel AR , Ritzel R, McCullough LD et al Microglia and ischemic stroke: a double-edged sword. International Journal of Physiology, Pathophysiology and Pharmacology 2013;5:73. 23750306 PMC3669736

[btae156-B14] Prinz M , Jung S, Priller J. Microglia biology: one century of evolving concepts. Cell 2019;179:292–311. 31585077 10.1016/j.cell.2019.08.053

[btae156-B15] Reddaway J , Richardson PE, Bevan RJ et al Microglial morphometric analysis: so many options, so little consistency. Front Neuroinform 2023;17:1211188. 37637472 10.3389/fninf.2023.1211188 PMC10448193

[btae156-B16] Sholl D. Dendritic organization in the neurons of the visual and motor cortices of the cat. J Anat 1953;87:387–406. 13117757 PMC1244622

[btae156-B17] Sierra A , Paolicelli RC, Kettenmann H. Cien años de microglía: milestones in a century of microglial research. Trends Neurosci 2019;42:778–92. 31635851 10.1016/j.tins.2019.09.004

[btae156-B18] Sipe G , Lowery R, Tremblay M-È et al Microglial p2y12 is necessary for synaptic plasticity in mouse visual cortex. Nat Commun 2016;7:10905–15. 26948129 10.1038/ncomms10905 PMC4786684

[btae156-B19] Takano T. Role of microglia in autism: recent advances. Dev Neurosci 2015;37:195–202. 25998072 10.1159/000398791

[btae156-B20] Tang Y , Le W. Differential roles of m1 and m2 microglia in neurodegenerative diseases. Mol Neurobiol 2016;53:1181–94. 25598354 10.1007/s12035-014-9070-5

[btae156-B21] Tynan RJ , Naicker S, Hinwood M et al Chronic stress alters the density and morphology of microglia in a subset of stress-responsive brain regions. Brain Behav Immun 2010;24:1058–68. 20153418 10.1016/j.bbi.2010.02.001

