# Supplementary material for: A model-based hierarchical Bayesian approach to Sholl analysis
Source: Bioinformatics. 2024 Mar 21;40(4):btae156. doi: 10.1093/bioinformatics/btae156 (PMC10985672; doi:10.1093/bioinformatics/btae156)
Supplement: btae156_Supplementary_Data [file btae156_supplementary_data.pdf]

Supplementary Materials for A Model-Based Hierarchical Bayesian Approach to Sholl Analysis

ERIK VONKAENEL[1], ALEXIS FEIDLER[2], REBECCA LOWERY[2], KATHERINE ANDERSH[2],TANZY LOVE[1], ANIA MAJEWSKA[2], MATTHEW N. MCCALL*[1,3]

[1]*Department of Biostatistics and Computational Biology, University of Rochester, NY 14642, USA,* [2]*Department of Neuroscience, University of Rochester, NY 14642, USA,* [3]*Department of Biomedical Genetics, University of Rochester, NY 14642, USA*

mccallm@gmail.com

1. Methods

1.1 *Existing Methods*

There are two primary avenues for analyzing Sholl data: transformation-based and summary-based methods. Transformation-based methods involve linearizing Sholl curves so that ordinary least squares can be applied, the most common being the *semi-log* and *log-log* methods. For the ith concentric circle, let x_i be the radius, A_i be the area, and y_i be the number of intersections. Then the semi-log regression model is given by $\log_{10}\left(\frac{y_i}{A_i}\right) = -\beta x_i + \varepsilon_i$, where $\varepsilon_i \sim N(0,1)$. Similarly, the log-log model is given by $\log_{10}\left(\frac{y_i}{A_i}\right) = -\beta \log_{10}(x_i) + \varepsilon_i$, where $\varepsilon_i \sim N(0,1)$. The parameter β is called Sholl's regression coefficient, which is often interpreted as the decay rate of the number of branches with distance from the soma (Sholl, 1953; Milošević and Ristanović, 2007).

These linearizations can be quite poor (Figure 1), typically resulting in the transformed Sholl curve oscillating about the fitted linear curve. Additionally, we commonly have access to many cell images, often in some nested hierarchical structure induced by experimental design, so a single linearization technique may not be appropriate for all available data. Even if a model is reasonably chosen, we are still limiting our analysis of very rich data to a single parameter model.

Another strategy involves reducing the Sholl curve into a summary statistic, which can be

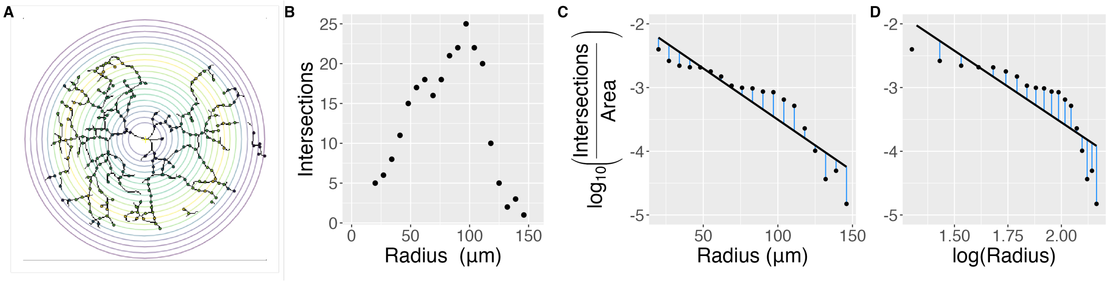

Fig. 1. Sholl analysis is performed on a skeletonized microglia. **A**: A skeletonized cell with concentric Sholl curves superimposed. **B**: The corresponding sholl curve. **C**: The semi-log transformed curve. **D**: The log-log transformed curve. In panels **C** and **D**, the black line is the fitted regression curve and the blue lines indicate the residuals.

passed to a hypothesis testing procedure. Some previously proposed Sholl curve summaries are:

- Branch Maximum: the maximum number of crossings across all radii
- Critical Value: the radius at which the maximum number of crossings is observed
- Schoenen Ramification Index: the branch maximum divided by the number of branches originating at the soma
- Area Under the Curve
- Full Width Half Max: the width of the curve at half the maximum number of crossings

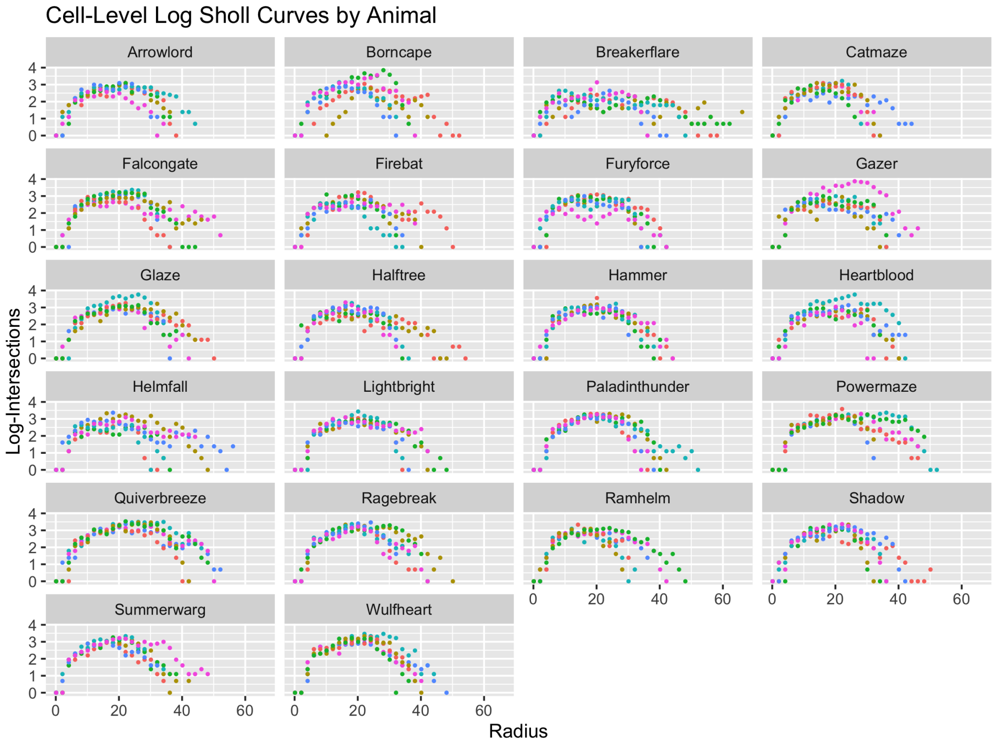

Fig. 2. Cell-level log-transformed Sholl curves are displayed for the ungrouped animal dataset (Section 3.1), where each facet corresponds to a difference animal. Our proposed model fits a piece-wise parabola to the log-mean process crossings, which captures the structure of the data directly.

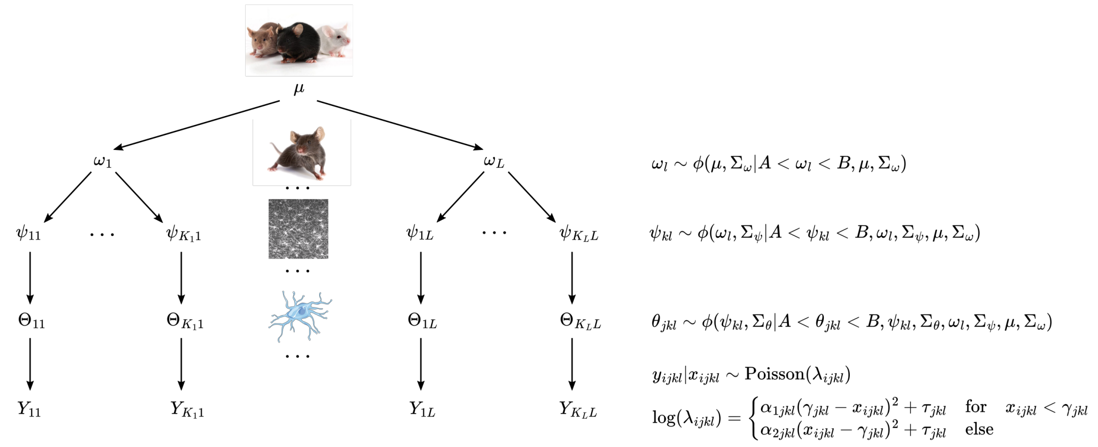

Fig. 3. Hierarchical structure for the ungrouped design. We assume parameters at any level are randomly sampled from the corresponding distribution in the next highest level. Here, $\phi(*)$ denotes the Gaussian distribution, μ denotes population-level parameters, ω denotes animal-level parameters, ψ denotes image-level parameters, θ denotes cell-level parameters, and Y denotes Sholl curve process crossings. For a given parameter $*$, Σ_* denotes variance parameters for the corresponding Gaussian. Gaussian priors are truncated via A and B to enforce the parameter constraints of the proposed model.

1.2 *Additional Specific Models*

The following models are specific operationalizations of the general formulation which are used in additional real data examples.

1.2.1 *Ungrouped Model* The hierarchical structure of this model is displayed in Figure 3. The population-level parameters seen in Figure 3 are defined as $\mu = (\mu_{\alpha_1}, \mu_{\alpha_2}, \mu_\gamma, \mu_\tau)$. Then for the lth animal, we define $\omega_l = (\omega_{\alpha_1 l}, \omega_{\alpha_2 l}, \omega_{\gamma l}, \omega_{\tau,l})$ and $\Sigma_\omega = \text{diag}(\sigma^2_{\omega\alpha_1}, \sigma^2_{\omega\alpha_2}, \sigma^2_{\omega\gamma}, \sigma^2_{\omega\tau})$. Parameters corresponding to the kth image of animal l are defined as $\psi_{kl} = (\psi_{\alpha_1 kl}, \psi_{\alpha_2 kl}, \psi_{\gamma kl}, \psi_{\tau kl})$ and $\Sigma_\psi = \text{diag}(\sigma^2_{\psi\alpha_1}, \sigma^2_{\psi\alpha_2}, \sigma^2_{\psi\gamma}, \sigma^2_{\psi\tau})$. In Figure 3, cell-level parameters for image k of animal l are vectorized as $\Theta_{kl} = (\theta_{1kl}, \ldots, \theta_{J_{kl}kl})$, so that parameters for cell j in image k of animal l are given by $\theta_{jkl} = (\theta_{\alpha_1 jkl}, \theta_{\alpha_2 jkl}, \theta_{\gamma jkl}, \theta_{\tau jkl})$ and $\Sigma_\theta = \text{diag}(\sigma^2_{\theta\alpha_1}, \sigma^2_{\theta\alpha_2}, \sigma^2_{\theta\gamma}, \sigma^2_{\theta\tau})$. We also vectorize the Sholl curve process crossings for cells in image k of animal l as $Y_{kl} = (\mathbf{y}_{1kl}, \ldots, \mathbf{y}_{J_{kl}kl})$, where $\mathbf{y}_{jkl} = (y_{1jkl}, \ldots, y_{N_{jkl}jkl})$ denote process crossings for cell j in image k of animal l.

We truncate normal distributions ϕ at each level of the hierarchy to match the parameter

space for the proposed model. The lower bound of the parameter space is $A = (-\infty, -\infty, 0, 0)$ and the upper bound is $B = (0, 0, \tilde{x}, \infty)$, where $\tilde{x}$ is the least upper bound on the support of the Sholl curves. As suggested in Gelman (2006), we assume a half-t prior on all standard deviation parameters.

1.2.2 *Modeling Groups at Different Levels* In this model, effects are incorporated at two separate levels of the hierarchy. The corresponding applied example contains population, genotype, animal, eye, and cell levels in that order. The full model is shown in Figure 4. At the genotype level, we add an effect for knockout (KO) as an additive term on the mean parameter for ξ^{KO}. Specifically,

$$\xi^{Geno} \sim \phi(\mu + I_{KO} \cdot b^{KO}, \Sigma_\xi | A < \xi^{Geno} < B, \mu + I_{KO} \cdot b^{KO}, \Sigma_\xi)$$

where

$$I_{Geno} = \begin{cases} 1 \text{ if } Geno = KO \\ 0 \text{ if } Geno = WT \end{cases}$$

The effect is given by

$$b^{KO} \sim \phi(\mu_{b^{KO}}, \Sigma_{b^{KO}} | (-\infty, -\infty, -\mu_\gamma, -\mu_\tau) < b^{KO} < (-\mu_{\alpha_1}, -\mu_{\alpha_2}, \infty, \infty), \mu_{b^{KO}}, \Sigma_{b^{KO}}).$$

In our real data example, cell-level curves correspond to either a control eye, or an eye subject to optical nerve crush injury. Each animal has a crush and control eye, so we model the effect of condition, and the interaction of condition and genotype at the cell level via

$$\begin{aligned} \theta_{jl}^{Cond/Geno} \sim \phi(&\omega_l^{Geno} + I_{Crush} \cdot b^{Crush} + I_{KO/Crush} \cdot b^{KO/Crush}, \Sigma_\theta | \\ &A < \theta_{jl}^{Cond/Geno} < B, \omega_l^{Geno}, b^{Crush}, b^{KO/Crush}, \Sigma_\theta, \\ &\xi^{Geno}, \Sigma_\omega, \mu, b^{KO}, \Sigma_\xi) \end{aligned}$$

where

$$I_{Crush} = \begin{cases} 1 \text{ if } Cond = Crush \\ 0 \text{ if } Cond = Control \end{cases}$$

and

$$I_{KO/Crush} = \begin{cases} 1 \text{ if } Cond = Crush \text{ and } Geno = KO \\ 0 \text{ else.} \end{cases}$$

The effect of condition is given by

$$b^{Crush} \sim \phi(\mu_{b^{Crush}}, \Sigma_{b^{Crush}} | A_{b^{Crush}} < b^{Crush} < B_{b^{Crush}}, \mu_{b^{Crush}}, \Sigma_{b^{Crush}})$$

where

$$A_{b^{Crush}} = \big(-\infty, -\infty, -\min(\Omega_\gamma), -\min(\Omega_\tau) \big)$$

$$B_{b^{Crush}} = \big(-\min(\Omega_{\alpha_1}), -\min(\Omega_{\alpha_2}), \infty, \infty \big)$$

and $\Omega_* = (\omega^{WT}_{*,1}, \dots, \omega^{WT}_{*,L_{WT}}, \omega^{KO}_{*,1}, \dots, \omega^{KO}_{*,L_{KO}})$.

The interaction effect is

$$b^{KO/Crush} \sim \phi(\mu_{b^{KO/Crush}}, \Sigma_{b^{KO/Crush}} | A_{b^{KO/Crush}} < b^{KO/Crush} < B_{b^{KO/Crush}}, \mu_{b^{KO/Crush}}, \Sigma_{b^{KO/Crush}})$$

where

$$A_{b^{KO/Crush}} = \Big(-\infty, -\infty, -\big\{ \min(\Omega_\gamma) + b_\gamma^{KO/Crush} \big\}, -\big\{ \min(\Omega_\tau) + b_\tau^{KO/Crush} \big\} \Big)$$
$$B_{b^{KO/Crush}} = \Big(-\big\{ \min(\Omega_{\alpha_1}) + b_{\alpha_1}^{KO/Crush} \big\}, -\big\{ \min(\Omega_{\alpha_2}) + b_{\alpha_2}^{KO/Crush} \big\}, \infty, \infty \Big).$$

As before, we assume half-t priors on all standard deviation parameters.

2. Simulation Details

Unless otherwise indicated, all simulation parameters are identical to the baseline scenario. At baseline, we simulate data using 5 animals per group and 10 cells per animal. For scenario 5, we double the cells per animal assumed at baseline. The baseline group-level variance is set as $\Sigma^{(1)} = \text{diag}(0.000025^2, 0.000025^2, 0.5^2, 0.05^2)$, while baseline variance parameters for all other levels are set as $\Sigma^{(i)} = \text{diag}(0.0001^2, 0.0001^2, 1, 0.1^2)$. For scenario 6, we set $\sigma_\tau^2 = 0.25^2$ at the animal-level, i.e. $\Sigma^{(2)} = \text{diag}(0.0001^2, 0.0001^2, 1, 0.25^2)$. Population level parameters are initialized as $\Theta^{(0)} = (-0.002, -0.002, 30, 2)$ for each scenario.

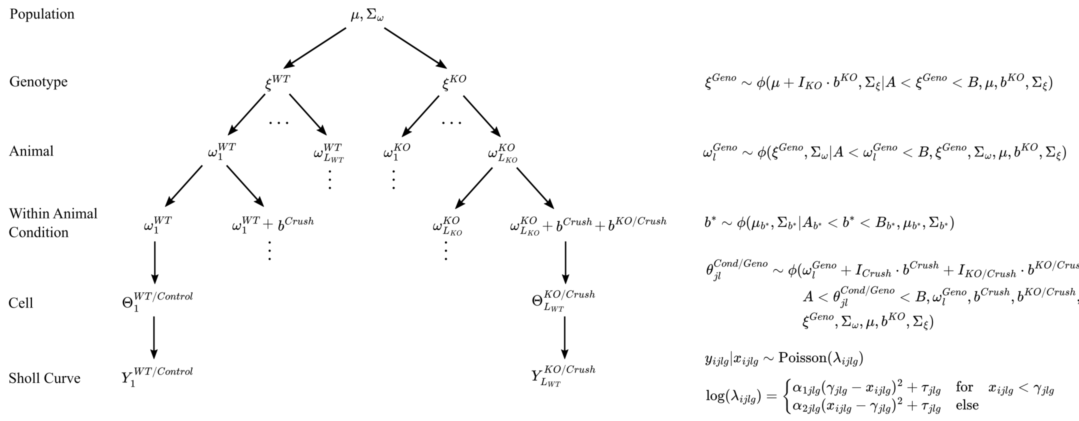

Fig. 4. Hierarchical structure for within and between animal effects. As before, all notation is shared with models displayed in Figures 1.2 and 2, except ξ denotes genotype-level parameters. Additionally, b^{KO} denotes the genotype level effect, b^{Crush} denotes the condition level effect, and $b^{KO/Crush}$ denotes the interaction effect between condition and genotype. I_* is an indicator variable equal to 1 for observations in group $*$, and 0 else.

50 datasets are simulated for each scenario, and both models are fit to each dataset. For each dataset, we simulate a seed for each of 4 chains which are all run in parallel. Then for each chain, a JAGS sampler was adapted for 5000 iterations, followed by a burn in of 15000 and 20000 iterations. Auto-correlation is alleviated by thinning, keeping a sample every 20 iterations.

To benchmark the proposed method, we first define the posterior probability that some effect b is less than 0 for simulation run i as $P_i(b < 0|*)$. Now define

$$\hat{p}_{<0} = \frac{\sum_{i=1}^{50} I\{\hat{P}_i(b < 0|*) > 0.95\}}{50}, \tag{2.1}$$

which is the proportion of simulation runs where the estimated posterior probability of some effect b having a negative sign is greater than 0.95. We estimate $\hat{P}_i(b < 0|*)$ as the proportion of MCMC samples for parameter b that fall below 0. We can similarly define $P_i(b > 0|*)$ and

$$\hat{p}_{>0} = \frac{\sum_{i=1}^{50} I\{\hat{P}_i(b > 0|*) > 0.95\}}{50}. \tag{2.2}$$

Across simulation runs, we expect $\hat{p}_{<0}$ (or $\hat{p}_{>0}$) to approach 1 as the relative strength of a negative (or positive) effect increases. Similarly, if there is no true effect, we expect both $\hat{p}_{<0}$ and

Scenario	Parameter	Effect		
		Condition	Side	Interaction
1	α_1	0	0	0
	α_2	0	0	0
	γ	0	0	0
	τ	0	0	0
2	α_1	0	0	0
	α_2	0	0	0
	γ	0	0	0
	τ	0.5	0	0
3	α_1	0	0	0
	α_2	0	0	0
	γ	0	0	0
	τ	0	-0.25	0
4	α_1	0	0	0
	α_2	0	0	0
	γ	0	0	0
	τ	0.5	-0.25	0.5
5	α_1	0	0	0
	α_2	0	0	0
	γ	0	0	0
	τ	0.5	-0.25	0.5
6	α_1	0	0	0
	α_2	0	0	0
	γ	0	0	0
	τ	0.5	-0.25	0.5

Table 1. Effects for each simulation scenario. We only consider effects on τ as it's the most relevant parameter for the purposes of this article.

$\hat{p}_{>0}$ to be approximately 0. Similar to the estimated power and FPR in frequentist simulations, $\hat{p}_{<0}$ and $\hat{p}_{>0}$ can be interpreted as the proportion of simulation runs where our decision criteria is met. Thus, we can add $\hat{p}_{<0}$ and $\hat{p}_{>0}$ to measure the probability of a false discovery when there is no true effect.

3. Additional Applied Examples

3.1 *Ungrouped Mouse Dataset*

Sections of mouse cortical tissue were generated and underwent histology for a microglia-specific marker. Images of microglia in the primary visual cortex were collected, and Sholl analysis was performed to assay the number of microglial processes at regular distance intervals from the cell soma. These data were plotted as Sholl curves to represent the overall morphological profile of

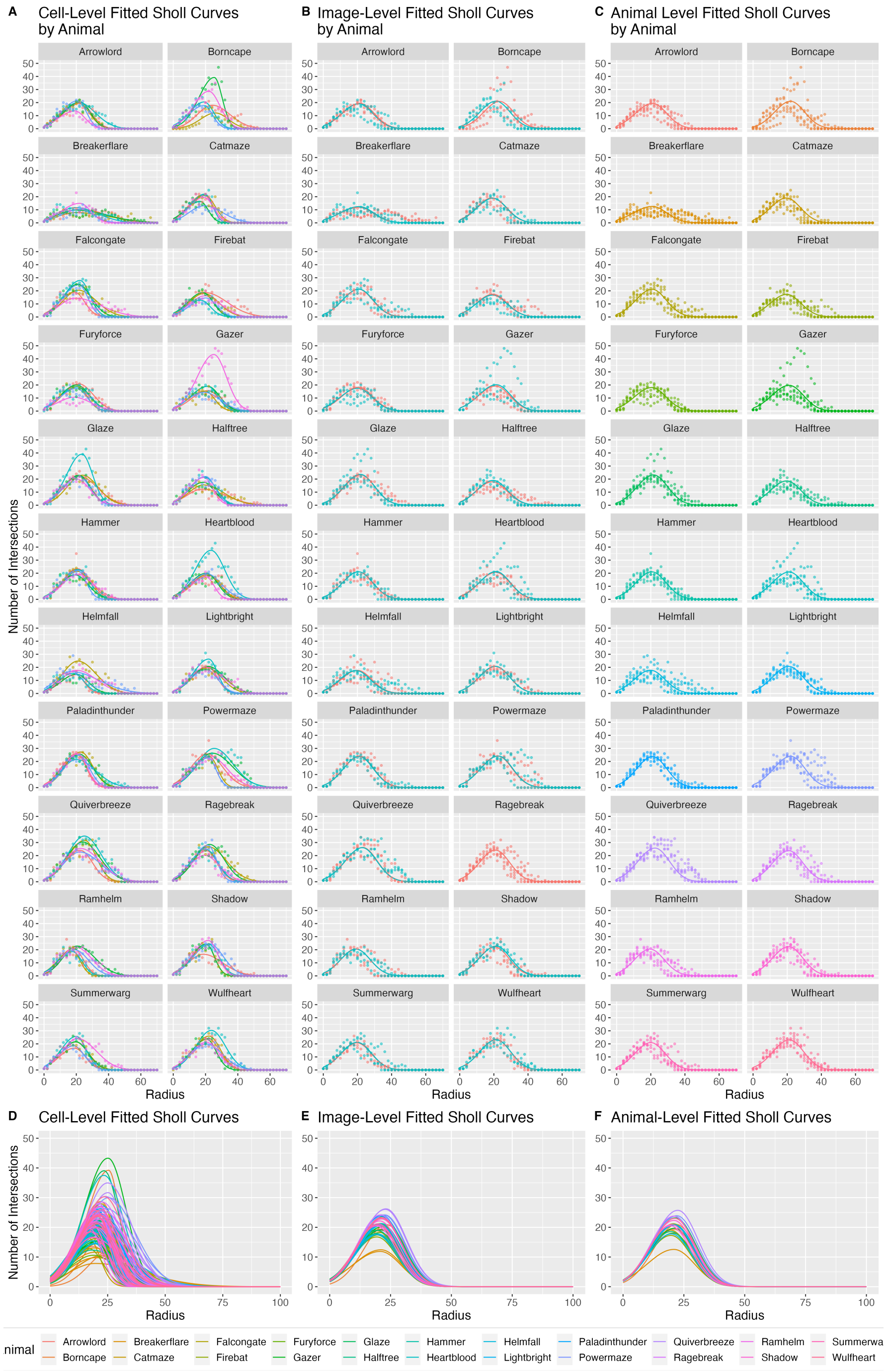

Fig. 5. Fitted curves at each level for the model discussed in Section 1.2. **A**: Cell-level fitted curves for each animal, where color indicates the cell. **B**: Image-level fitted curves for each animal, where color indicated image. **C**: Animal-level fitted curves. **D**: All cell-level fitted curves displayed in panel A, superimposed to show the cell-level variation. **E**: All image-level fitted curves displayed in panel B, superimposed to show the image-level variation. **F**: All animal-level fitted curves displayed in panel C, superimposed to show the animal-level variation.

individual microglia. Original analysis detected a range of Sholl curve profiles at the level of individual cells which were used to generate animal level aggregate Sholl curves.

Figure 5 shows the fitted curves at each level of the model hierarchy. In this example, we are primarily interested in the model's ability to capture the possible range of Sholl curves at any level of the hierarchy. This desired flexibility is particularly apparent at the cell level, where Sholl curves can vary greatly within an animal. For example, we see the model has no issues capturing the curve with abnormally large branch maximum associated with Gazer. There is not much variation between images within an animal, which isn't surprising because images are taken of adjacent areas in the same brain region. The model is able to capture an overall animal level curve quite well, while also allowing for natural variations between animals.

3.2 *GPNMB Knockout Dataset*

This data set was generated to investigate the effect of the loss of transmembrane glycoprotein NMB (GPNMB) on the microglial response to an optic nerve crush (ONC) injury. GPNMB can work to reduce inflammation and is highly expressed in microglia, so the presence or absence of GPNMB may influence the role of microglia in the retina following ONC injury. An ONC injury was performed on a pilot cohort of 9 mice. Mice had either wildtype expression of GPNMB or a genetic knockout. For each animal, crush was performed on one eye and the contralateral eye underwent a sham injury which served as an intra-animal control. Retinas were collected 7 days after injury. Retinas were stained for microglia-specific markers (including ionized calcium-binding adapter molecule 1 (Iba1) and the ganglion cell layer/inner plexiform layer was imaged using confocal microscopy at 40X magnification. Z-stacks were projected in the z dimension and binarized with a set threshold. Sholl analysis was performed on individual microglia and, similar to above, analysis was performed by constructing animal level aggregate Sholl curves. An ANOVA with repeated measures was conducted on both the branch maximum and critical value to test

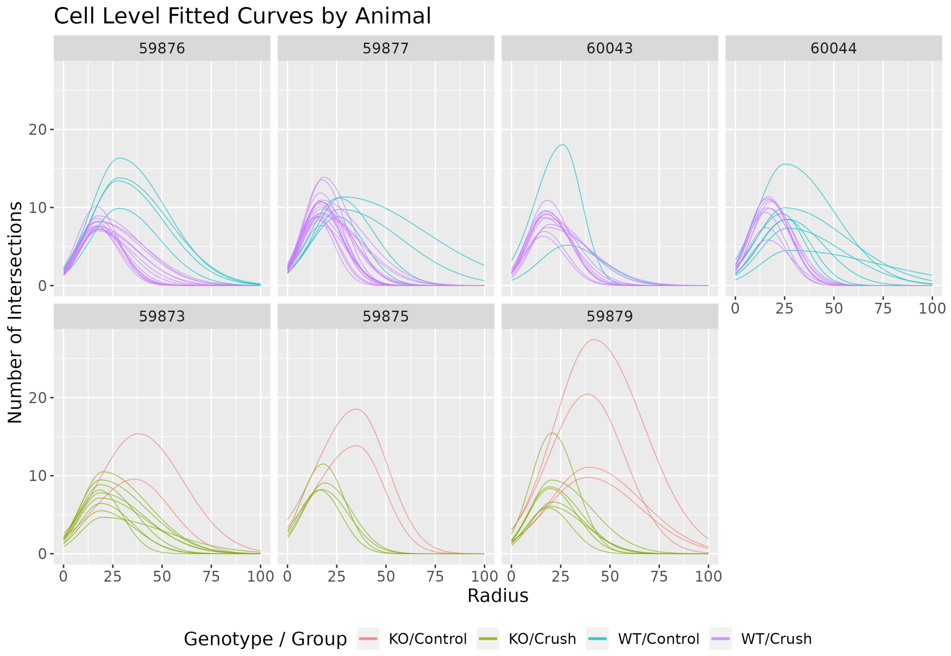

Fig. 6. Cell-level fitted curves faceted by animal obtained by fitting the model discussed in Section 1.2.2 to the GPNMB knockout dataset. An animal is either wild-type (WT), or has gene GPNMB knocked out (KO). Cells are associated with either an eye subject to optical nerve crush injury, or control. Each animal has both a crush eye and a control eye.

the differences between genotype, condition, and interaction. Results of this analysis are shown in Table 2.

Cell-level fitted curves, separated by animal are displayed in Figure 6. Figure 7 shows 95% credible intervals for effects on genotype, condition, and interaction, superimposed over approximate posterior distributions. As with the MD/ND example, we report the estimated posterior probability each effect is less than 0 in Table 3. Using an 0.95 cutoff as before, we see the proposed method and two-way ANOVA detected similar effects on the branch maximum, while the proposed method also detects genotype and interaction effects on the critical value. Additionally, our method offers increased granularity when differences between curves are not obvious. Unlike the MD/ND example, visual differences between fitted curves in Figure 6 are not limited to these two summaries. Using our method, we are able to quantify these differences by leveraging α_1 and α_2,

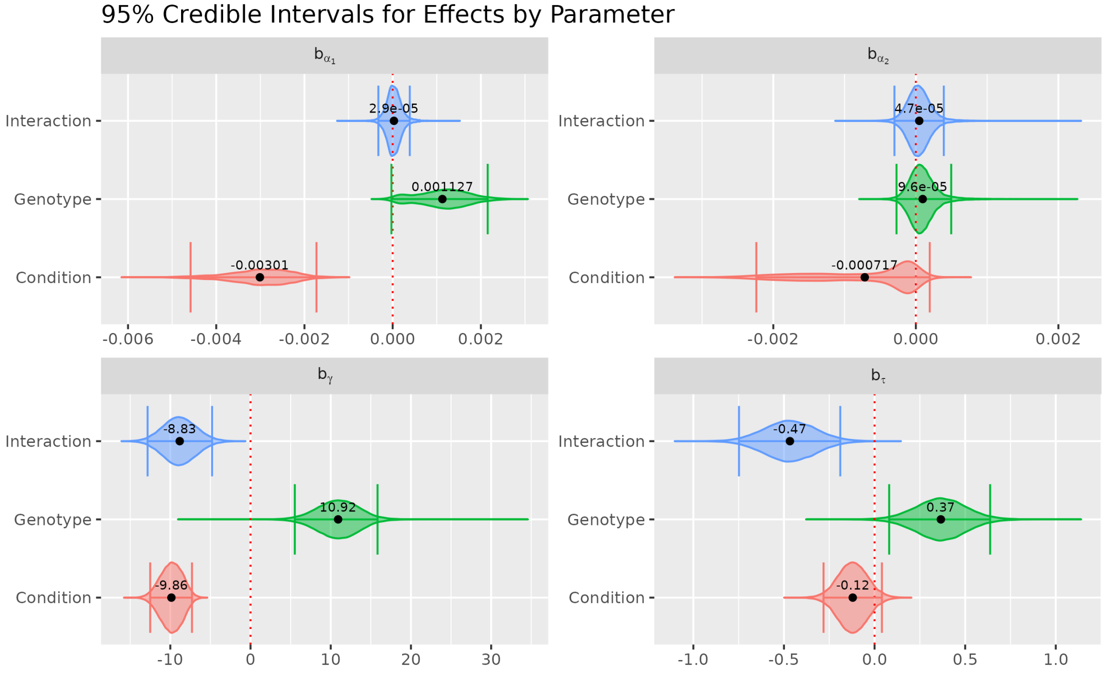

Fig. 7. 95% credible intervals for each effect in model 3, fitted to the GPNMB knockout dataset. Credible intervals are computed as the highest density posterior interval. Credible intervals are superimposed over the approximate posterior distributions obtained via MCMC. Estimated posterior means are represented by black dots with point estimates displayed above. The dotted red line is fixed at 0.

rather than only relying on the curve maximum. Specifically, we detect a negative condition effect on α_1, meaning crush curves have steeper growth states than control curves. Though we only report effects here, we also have the option to investigate parameters, associated variance terms, and combinations of parameters (such as the y-intercept) at each level of the model hierarchy, providing a rich toolbox for investigating subtle curve differences.

3.3 *MCMC Sampling Procedures*

3.3.1 *Ungrouped Mouse Dataset* We sample from the posterior using the JAGS implementation of MCMC, running 4 chains in parallel. For each chain, we we allow the sampler to adapt for 5000 iterations, followed by a 50000 iteration burn-in. The sampler is then run for 150000 iterations.

Response	Effect	F	p-value
Branch Maximum	Genotype	14.05	0.01
	Condition	34.62	0.00
	Interaction	10.89	0.02
Critical Value	Genotype	14.05	0.01
	Condition	34.62	0.00
	Interaction	10.89	0.02

Table 2. Two-way ANOVA with repeated measures fit to the branch maximum and critical value of the truncated GPNMB knockout data

Parameter	$\hat{P}(\text{Effect} < 0)$		
	Condition	Genotype	Interaction
α_1	1.000	0.020	0.442
α_2	0.891	0.315	0.402
γ	1.000	0.000	1.000
τ	0.931	0.007	0.999

Table 3. Estimated posterior probability of a negative effect for each parameter in the GPNMB knockout model. Quantities are estimated as the proportion of MCMC samples that fall below 0.

Autocorrelation is alleviated by thinning, keeping a sample every 50 iterations.

3.3.2 *MD/ND Dataset* For each of 4 chains, we adapted a JAGS sampler for 5000 iterations, followed by a 50000 iteration burn in before running the sampler for 150000 iterations. Autocorrelation is alleviated by thinning, keeping a sample every 50 iterations.

3.3.3 *GPNMB Knockout Dataset* As before, the model is fit using a JAGS sampler and 4 chains, each of which are adapted with 10000 iterations. A burn-in of 250000 iterations was performed before obtaining 500000 samples. Auto-correlation is alleviated by thinning, keeping a sample every 50 iterations.

References

Gelman, Andrew. (2006). Prior distributions for variance parameters in hierarchical models (comment on article by browne and draper). *Bayesian analysis* **1**(3), 515–534.

Milošević, Nebojša T. and Ristanović, Dušan. (2007). The sholl analysis of neuronal cell images: Semi-log or log–log method? *Journal of Theoretical Biology* **245**(1), 130–140.

Sholl, D.A. (1953). Dendritic organization in the neurons of the visual and motor cortices of the cat. *Journal of Anatomy*, 387–406.
